# Hypothalamic involvement in premature aging laminopathies

**DOI:** 10.1186/1750-1172-10-S2-O6

**Published:** 2015-11-11

**Authors:** Claudia Cavadas

**Affiliations:** 1 CNC – Center for Neuroscience and Cell Biology, University of Coimbra, Coimbra, Portugal, Faculty of Pharmacy, University of Coimbra, Coimbra, Portugal

## 

Caloric restriction (CR), the reduced intake of calories without malnutrition, extends lifespan of many organisms, from yeast to mammals, and delays the progression of age-related diseases. Evidence show that hypothalamus is a crucial brain region for the progress of whole-body aging[1] and the beneficial effects induced by CR are regulated by nutrient-sensing neurons located in the hypothalamus[2]. Although CR's beneficial effects in delaying human aging are promising, its application for long periods is very difficult to maintain and not feasible to apply to fragile children with progeria. To overcome this problem, the induction of protective endogenous mechanisms, or pharmacological agents, could theoretically be used to mimic the beneficial effects of CR without its discomfort. Our group showed that hypothalamus of Zmpste24-/- mouse has lower levels of Neuropeptide Y, comparing to wild-type animals. Moreover, they showed that targeting the Neuropeptide Y system in hypothalamus, as a CR mimetic strategy, delays or reverts some ageing features of Zmpste24-/- mice. Further studies are needed to confirm this innovative approach and if it could be translational to progeria children.
